# A TRANSPARENT TESTA Transcriptional Module Regulates Endothelium Polarity

**DOI:** 10.3389/fpls.2019.01801

**Published:** 2020-02-06

**Authors:** Olivier Coen, Jing Lu, Wenjia Xu, Stéphanie Pateyron, Damaris Grain, Christine Péchoux, Loïc Lepiniec, Enrico Magnani

**Affiliations:** ^1^Institut Jean-Pierre Bourgin, INRA, AgroParisTech, CNRS, University of Paris-Saclay, Versailles, France; ^2^École Doctorale 567 Sciences du Végétal, University Paris-Sud, University of Paris-Saclay, Orsay, France; ^3^TranscriptOmic Platform of IPS2, Institute of Plant Sciences Paris Saclay IPS2, CNRS, INRA, Université Paris-Sud, Université Evry, Université Paris-Saclay, Orsay, France; ^4^INRA, Génétique Animale et Biologie Intégrative, Jouy-en-Josas, France

**Keywords:** seed coat, endothelium, polarity, TRANSPARENT TESTA, fertilization

## Abstract

Seeds have greatly contributed to the successful colonization of land by plants. Compared to spores, seeds carry nutrients, rely less on water for germination, provide a higher degree of protection against biotic and abiotic stresses, and can disperse in different ways. Such advantages are, to a great extent, provided by the seed coat. The evolution of a multi-function seed-coat is inheritably linked to the evolution of tissue polarity, which allows the development of morphologically and functionally distinct domains. Here, we show that the endothelium, the innermost cell layer of the seed coat, displays distinct morphological features along the proximal-distal axis. Furthermore, we identified a TRANSPARENT TESTA transcriptional module that contributes to establishing endothelium polarity and responsiveness to fertilization. Finally, we characterized its downstream gene pathway by whole-genome transcriptional analyses. We speculate that such a regulatory module might have been responsible for the evolution of morphological diversity in seed shape, micropylar pore formation, and cuticle deposition.

## Introduction

**Figure 1:**
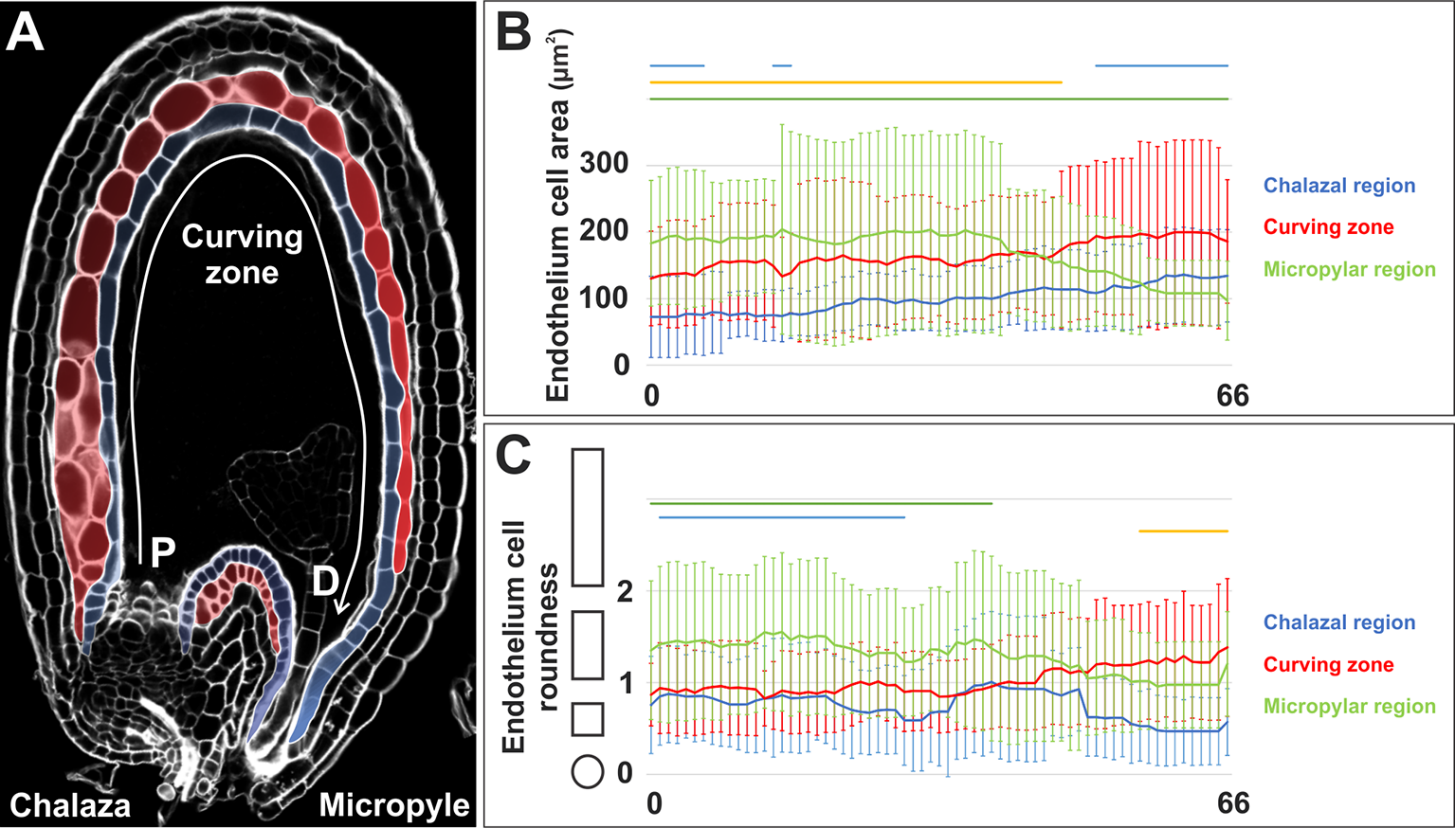
The endothelium proximal-distal polarity axis. **(A)** Longitudinal mid-plane of a wild type seed at 6 DAF. The endothelium is highlighted in blue whereas ii1’ and ii1’’ cells are highlighted in red. P, proximal; D, distal. **(B**, **C)** Average endothelium cell area in µm^2^ **(A)** and cell roundness **(B)** (see *Methods*) along the proximal-distal axis of the chalazal, curving zone, and micropylar regions (arbitrarily divided in 67 points) as observed in longitudinal mid-planes of wild type seeds at 4 DAF. The shapes on the left of the graph in panel B exemplify how cell shape changes along the *y*-axis. Lines at the top of the graph indicate regions of statistically significant difference between chalazal region and curving zone (green), chalazal and micropylar regions (yellow), and curving zone and micropylar region (red) (two-tailed Student’s *t*-test; P < 0.05). Error bars indicate standard deviation (n = 33). Ecotype Ws.

The seed coat consists of cell layers that surround, nourish, protect, and facilitate the dispersal of the fertilization product(s) (Coen and Magnani, 2018). As a whole, the seed coat is a highly polar structure displaying morphologically and functionally distinct domains responsible for different seed functions (Haughn and Chaudhury, 2005; Coen and Magnani, 2018). Along the seed medial-lateral polarity axis, different cell layers follow unique differentiation pathways. Furthermore, cell-patterning along the proximal-distal axis defines the chalazal and micropylar regions (**Figure 1A**). Finally, integument number and cell thickness deeply affect seed curvature along the adaxial–abaxial axis.

In Arabidopsis, the seed coat comprises an outer (oi) and an inner (ii) integument (Schneitz et al., 1995). Both ii and oi initiate in the ovule as two-cell layered primordia (ii1 or endothelium, ii2, oi1, and oi2) that grow by anticlinal cell divisions to surround the female gametophyte. At the end of ovule development, periclinal cell divisions of the endothelium, the innermost integument cell layer, give rise to one or two sub-epidermal cell layers (ii1’ and ii1’’) (**Figure 1A**) (Schneitz et al., 1995; Debeaujon et al., 2003; Coen et al., 2017). Furthermore, a fraction of ovules displays additional sub-epidermal cell strings (oi1’), of chalazal origin, in between oi1 and oi2 (Fiume et al., 2017a). Double fertilization of the female gametophyte gives rise to embryo and endosperm and marks the transition from ovule to seed (Schneitz et al., 1995). The seed coat does not actively participate in the fertilization process but undergoes cell expansion and differentiation in coordination with the fertilization products (Ingram, 2010).

The endothelium lies at the interface between maternal tissues and fertilization products and displays unique biological properties. On the one hand, it shows remarkable developmental plasticity in interplaying with the endosperm to orchestrate seed growth (Ingram, 2010). On the other hand, it becomes a highly differentiated tissue. A cutin-based apoplastic barrier is deposited on the adaxial side of the endothelium, toward the endosperm, during ovule development, and in response to fertilization (De Giorgi et al., 2015; Loubery et al., 2018; Coen et al., 2019). Furthermore, fertilization triggers the accumulation in the endothelium of proanthocyanidins (PAs), flavonoid compounds that give the characteristic brown color to Arabidopsis seeds (Lepiniec et al., 2006). Genetic screens have identified several mutants defective in PA accumulation, collectively named *transparent testa* (*tt*). *TT* loci are involved in different aspects of tannin deposition: biosynthesis, transport, and regulation (Koornneef, 1990; Lepiniec et al., 2006). A handful of genes have been also found to regulate ii development. The MADS box transcription factor TT16 not only regulates PAs accumulation and cutin deposition but also endothelium cell expansion and orientation (Nesi et al., 2002; Ehlers et al., 2016; Coen et al., 2017; Coen et al., 2019). Furthermore, TT16 works redundantly with another MADS box transcription factor, SEEDSTICK (STK), to initiate endothelium periclinal cell divisions (Mizzotti et al., 2012; Coen et al., 2017) and oppositely to SHATTERPROOF 1 and 2 MADS box transcription factors to establish ii1’ proximal-distal polarity (Ehlers et al., 2016). The WRKY transcription factor TRANSPARENT TESTA GLABRA 2 (TTG2) coordinates integuments and endosperm growth (Garcia et al., 2005). *ttg2* mutant seeds show premature arrest of endosperm development and reduced seed size. Finally, the TT1 C2H2 zinc-finger transcription factor plays a role in PAs deposition and cuticle biosynthesis but there have been conflicting reports concerning its role in regulating endothelium cell shape (Sagasser et al., 2002; Debeaujon et al., 2003; Appelhagen et al., 2011; Coen et al., 2019).

Here we show that endothelium development is modulated along the proximal-distal axis. Our expression and genetic analyses indicate a role for TT1 in endothelium and ii1’ polar cell patterning. Furthermore, we tested TT1 genetic interaction with all known regulators of endothelium development and placed TT1 downstream of TT16 in the development of the proximal region of the inner integument. Finally, transcriptomic analyses of an inducible form of TT1 revealed its downstream target genes.

## Methods

### Plant Material

*Arabidopsis thaliana* plants of ecotype Columbia (Col-0) or Wassilewskija (Ws-2) were used as wild type controls as appropriate. The *tt16-1* mutant was isolated in the Ws-2 accession and then backcrossed to the Col-0 accession more than three times (Nesi et al., 2002; Xu et al., 2016). *tt1-3, stk-2*, *shp1-1;shp2-1*, and *map18*/*pcap2* mutants are in the Col-0 accession (Liljegren et al., 2000; Pinyopich et al., 2003; Appelhagen et al., 2011; Kato et al., 2019). *tt1-4* mutant is in the Ws-2 accession (Brunaud et al., 2002). *ProML1:gML1-mCitrine;ml1-3* line is in the Col-0 accession (Meyer et al., 2017).

Days after flowering were counted starting from the emergence of the pistil from closed flowers; 0 DAF equals stage 3-V of ovule development (Schneitz et al., 1995).

### Transgenic Plants

The *Agrobacterium tumefaciens* strain C58C1 was used to stably transform Arabidopsis plants using the floral dip method (Clough and Bent, 1998). Transformants were selected on MS medium containing hygromycin (50 mg L^−1^) and subsequently transferred to soil for further characterization.

### Expression Analysis by Quantitative PCR

Ovules and seeds used for total RNA extraction were frozen in liquid nitrogen immediately after harvest and stored at −80°C prior to extraction. Four independent biological samples were used for each analysis. Each replicate comprised the content in ovules/seeds of 10 to 15 pistil/siliques. Total RNA was extracted using the RNeasy Mini kit (Qiagen), including RNase-Free DNase Set (Qiagen) treatment during washing, according to the manufacturer’s instructions, and subsequently stored at −80°C. The Superscript Reverse Transcriptase II kit (Invitrogen) was used to generate cDNA from 1 µg of total RNA. Each cDNA sample was diluted 1:125 in water. Quantitative PCRs were performed with the SYBR Green kit (Bio-Rad) on a Bio-Rad CFX real-time PCR machine. For each reaction, 4.4 µL of diluted cDNA were added to 5 µl of SYBR Green and to 0.3 µl of each primer (10 µM) (**Supplemental Table 2**). Melt curves have been performed and primer efficiency has been tested (primers with efficiency between 85% and 100% have been used in this study). Expression levels were first normalized by the geometrical mean of the expression levels of four reference genes (*GAPDH*, *AT4G12590*, *AT4G02080*, and *AT3G25800*) (Dekkers et al., 2012), and subsequently normalized by the expression level of the adequate control. Means and standard deviations were calculated from four independent biological samples.

### Cloning and Construction

PCR amplification of *3kbProTT1:gTT1* was performed from Arabidopsis (Col-0) genomic DNA (https://www.ncbi.nlm.nih.gov/gene/840386 ) using the gene-specific primers (5′- GGGGACAAGTTTGTACAAAAAAGCAGGCTCTAACCATTTGCTTGTGTCAACA -3′) and (5′- GGGGACCACTTTGTACAAGAAAGCTGGGTCAAAAAACAAAGTCTCGGAGACAG -3′) carrying the *attB1* and *attB2* Gateway recombination sites. *3kbProTT1:gTT1* was recombined into the *pDONR207* vector (Gateway recombination). Compared to what annotated on the TAIR website (www.arabidopsis.org), the *3kbProTT1:gTT1* DNA fragment misses an adenine in position −1250 of the promoter and the CATATATATATATATATATATATATATATATATA sequence in position 605–638 of the intron. PCR amplification of *gTT1* was performed using the gene-specific primers (5′- GGGGACAAGTTTGTACAAAAAAGCAGGCTCAATGGAGTCACCACCACTATACGAGA -3′) and (5′- GGGGACCACTTTGTACAAGAAAGCTGGGTCAAAAAACAAAGTCTCGGAGACAGAT -3′) carrying the *attB1* and *attB2* Gateway recombination sites. For GR-inducible analyses, the *gTT1* sequence was recombined (Gateway recombination) into the *pR1R2ΔGR* binary vector (Baudry et al., 2004).

### Microscopy

Prior to microscopy analyses, siliques were dissected and septums (containing seeds) were harvested.

Calcofluor M2R white (fluorescent brightener 28; Sigma Aldrich) analyses were conducted as previously described (Coen et al., 2019). GFP and citrine expressing lines were analyzed 1 hour after mounting in a Propidium iodide (100 µg mL^−1^), sucrose (7%) solution, as previously described (Figueiredo et al., 2016). mPS-PI samples were prepared as previously described (Xu et al., 2016). Transmission electron microscopy analyses were conducted as previously described (Coen et al., 2019).

Modified pseudo-Schiff propidium iodide (mPS-PI) or calcofluor stained and GFP or citrine fluorescent samples were analyzed with a Leica TCS-SP5 or Leica TCS-SP8 spectral confocal laser scanning microscope (Leica Microsystems). For TEM, samples were examined with Hitachi HT7700 electron microscope operated at 80 kV (Elexience—France), and images were acquired with a charge-coupled device camera (AMT). For confocal microscopy, pictures showing mid-plane longitudinal sections of seeds were captured when possible. Otherwise, three dimensional z-stacks were acquired, and the mid-plane longitudinal sections were obtained with the Volume Viewer plugin of the Image J software (Schneider et al., 2012).

### Quantitative Morphological Analyses

Quantitative analyses of cell area and roundness were conducted as previously described (Coen et al., 2017). Ratio between endothelium anticlinal and periclinal cell length was obtained by assimilating endothelium cells to rectangles (in first approximation), and calculating their growth polarity (GP) as $\mathrm{GP}=\frac{1}{2}(\frac{P}{L}-2)$ where *P* and *L* represent perimeter and periclinal length of the cell, respectively. In the case of a perfect rectangle, GP represents the ratio between anticlinal and periclinal lengths.

### Transcriptome Studies

Six Col-0 and *Pro35S:gTT1-GR* main inflorescences at stage 6.50 (Boyes et al., 2001) were harvested and immersed in a Silwet 0.005% and 50 μM cycloheximide (CHX) solution, vacuum treated for 30 minutes, and incubated for 1 hour at room temperature. A dexamethasone (DEX) solution in ethanol was added to three Col-0 and *Pro35S:gTT1-GR* CHX-treated inflorescences to a final concentration of 50 µM. The same amount of ethanol without DEX was added to the remaining three Col-0 and *Pro35S:gTT1-GR* CHX-treated inflorescences. Finally, the inflorescences were vacuum treated for 30 minutes, incubated for 1 hour at room temperature, and frozen individually in liquid nitrogen. Total RNA was extracted using Ambion Mirvana miRNA kit, followed by the Ambion TURBO DNAse kit, according to the supplier’s instructions. The RNA integrity number was higher than eight. Microarray analysis was carried out using the CATMAv7 array (Lurin et al., 2004) based on AGILENT technology. Three independent biological replicates were produced. For each comparison, one technical replicate with fluorochrome reversal was performed for each biological replicate (i.e. four hybridizations per comparison). The labeling of cRNAs with Cy3-dUTP or Cy5-dUTP was performed as described in Two-Color Microarray-Based Gene Expression Analysis Low Input Quick Amp Labeling manual (Agilent Technologies, Inc.). The hybridization and washing were performed according to Agilent Microarray Hybridization Chamber User Guide instructions (Agilent Technologies, Inc.). Two micron scanning was performed with InnoScan900 scanner (Innopsys, Carbonne, France) and raw data were extracted using Mapix software (Innopsys, Carbonne, France).

### Statistical Analysis of Microarray Data

For each array, the raw data comprised the logarithm of median feature pixel intensity at wavelengths 635 nm (red) and 532 nm (green). For each array, a global intensity-dependent normalization using the loess procedure (Yang et al., 2002) was performed to correct the dye bias. The differential analysis is based on the log-ratios averaging over the duplicate probes and over the technical replicates. Hence the numbers of available data for each gene equals the number of biological replicates and are used to calculate the moderated t-test (Smyth, 2004). Under, the null hypothesis, no evidence that the specific variances vary between probes is highlighted by Limma and consequently the moderated t-statistic is assumed to follow a standard normal distribution. To control the false discovery rate, adjusted p-values found using the optimized FDR approach of (Storey and Tibshirani, 2003) were calculated. We considered as being differentially expressed the probes with an adjusted p-value ≤ 0.05. The analysis was done with the R software (http://www.R-project.org). The function SqueezeVar of the Limma library was used to smooth the specific variances by computing empirical Bayes posterior means. The library kerfdr was used to calculate the adjusted p-values.

### Gene Ontology Analyses

Gene ontology annotation analyses were conducted on the TAIR website (www.arabidopsis.org). GO enrichment analyses were conducted using the gene ontology enrichment analysis and visualization tool (Eden et al., 2009).

### Data Deposition

Microarray data from this article were deposited in the international repository GEO, Gene Expression Omnibus (Edgar et al., 2002), accession number: GSE 134014. All steps of the experiment, from growth conditions to bioinformatic and statistical analyses, were detailed in CATdb (Gagnot et al., 2008) (Project: RA15-05_TT1) according to the “Minimum Information About a Microarray Experiment” standards.

### Accession Numbers

Sequence data from this article can be found in the GenBank/EMBL data libraries under the following accession numbers: *TT16* (AT5G23260), *TT1* (AT1G34790), *SHP1* (AT3G58780), *SHP2* (AT2G42830), *STK* (AT4G09960), *ML1* (AT4G21750), *PDF2* (AT4G04890), *CR4* (AT3G59420), *TT2* (AT5G35550), *TT8* (AT4G09820), *TTG1* (AT5G24520), *TTG2* (AT2G37260)*, FIE* (AT3G20740), and *MSI1* (AT5G58230). **Supplemental Table 1** lists the accessions numbers for the genes detected by microarray analyses.

## Results

### The Endothelium Displays a Proximal-Distal Polarity Axis

To determine if the endothelium displays morphological differences along the proximal distal axis (**Figure 1A**), we analyzed seeds using the modified pseudo-Schiff propidium iodide (mPS-PI) and quantified cellular parameters in the chalazal, curving zone and micropylar regions (**Figure 1A**, see *Methods*). The chalazal region of the endothelium was characterized by smaller and rounder cells, when compared to the other regions (**Figures 1B, C**). Micropylar endothelium cells changed from relatively large and elongated to relatively small and round along the proximal-distal axis (**Figures 1B, C**). Finally, the endothelium curving zone showed cellular parameters intermediate between the chalazal and micropylar regions (**Figures 1B, C**). This analysis indicate that the endothelium displays a proximal distal polarity axis.

### TRANSPARENT TESTA 1 Regulates the Polarity of Endothelium Cell Expansion

**Figure 2:**
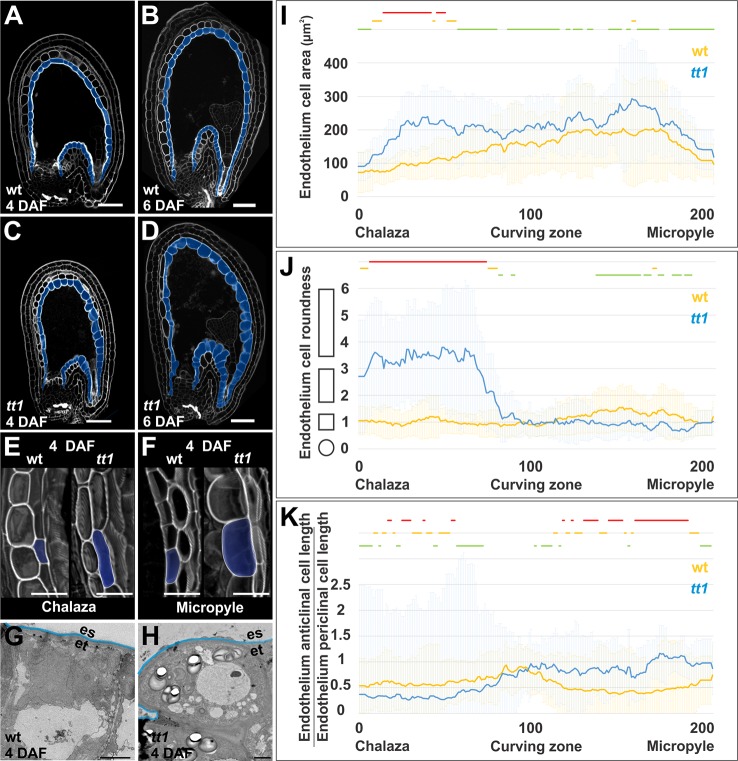
TT1 regulates endothelium cell expansion. **(A**–**D)** Longitudinal mid-planes of wild type (wt) and *tt1-4* seeds at 4 and 6 DAF, imaged using the mPS-PI technique. The endothelium is highlighted in blue. Ecotype Ws. Bars = 50 µm. **(E**, **F)** Longitudinal planes of three-dimensional reconstructed endothelium cells at the chalazal **(E)** and micropylar **(F)** region of wild type (wt) and *tt1-4* seeds at 4 DAF, imaged using the mPS-PI technique. One endothelium cell per sample is highlighted in blue. Ecotype Ws. Bars = 25 µm. **(G**, **H)** Transmission electron microscopy images of wild type (wt) **(G)** and *tt1-3* **(H)** endothelium cells at the micropylar region at 4 DAF. The interface between endosperm (es) and endothelium (en) is marked in blue. Ecotype Col-0. Bars = 25 µm. **(I**–**K)** Average endothelium cell area in µm^2^ **(I)**, endothelium cell roundness **(J)**, and ratio between endothelium anticlinal and periclinal cell length **(K)** (see *Methods*) along the seed coat proximal-distal axis (arbitrarily divided in 201 points) as observed in longitudinal mid-planes of wild type (wt, blue) and *tt1-4* (yellow) seeds at 4 DAF. The shapes on the left of the graph in panel J exemplify how cell shape changes along the *y*-axis. Lines at the top of the graph indicate regions of statistically significant difference between wild type and *tt1-4* (two-tailed Student’s *t*-test; green line: P < 0.05, orange line: P < 0.001, red line: P < 0.00001). Error bars indicate standard deviation (wild type n = 33, *tt1-4* n = 20). Ecotype Ws.

*TT16* and *TT1* are the only two genes that have been found to regulate endothelium cell patterning (Nesi et al., 2002; Sagasser et al., 2002; Ehlers et al., 2016; Coen et al., 2017). Nevertheless, the role of *TT1* in endothelium development is still controversial. Qualitative analyses of endothelium cell morphology in *tt1* mutant seeds by differential interference contrast microscopy gave conflicting results. *tt1* endothelium cells have been indeed reported as irregular in shape (Sagasser et al., 2002) or wild type looking (Appelhagen et al., 2011). To test if TT1 plays a role in endothelium cell patterning, we analyzed *tt1* mutant ovules and seeds by mPS-PI or calcofluor staining imaging techniques as well as by transmission electron microscopy. Whereas *tt1* and wild type ovules appeared indistinguishable, longitudinal mid-planes of *tt1* seeds at 4 DAF exhibited anticlinally over-elongated endothelium cells at the chalazal region and periclinally over-expanded endothelium cells on their adaxial side, leaning on the embryo, at the micropylar region, when compared to the wild type (**Figures 2A, C, G, H**, and **Supplemental Figure 1**). At 6 DAF, such *tt1* phenotypes were more pronounced (**Figures 2B, D–F**). To thoroughly characterize the effect of the *tt1* mutation on endothelium cell expansion, we quantified cell area and circularity along the endothelium proximal-distal axis in wild type and *tt1* seeds at 4 DAF. Overall, *tt1* endothelium cells exhibited an increased cell area when compared to the wild type (**Figure 2I**). Moreover, their shape appeared more elongated in the chalazal region and rounder in the micropylar region than in the wild type (**Figure 2J**). To examine the polarity of endothelium cell expansion, we calculated the ratio between anticlinal and periclinal cell length of wild type and *tt1* endothelium cells approximated to rectangles (see *Methods*). Whereas the periclinal cell length was on average greater than the anticlinal cell length all along the wild type endothelium, *tt1* seeds exhibited anticlinally elongated cells in a sub-domain of the micropylar region (**Figure 2K**).

**Figure 3:**
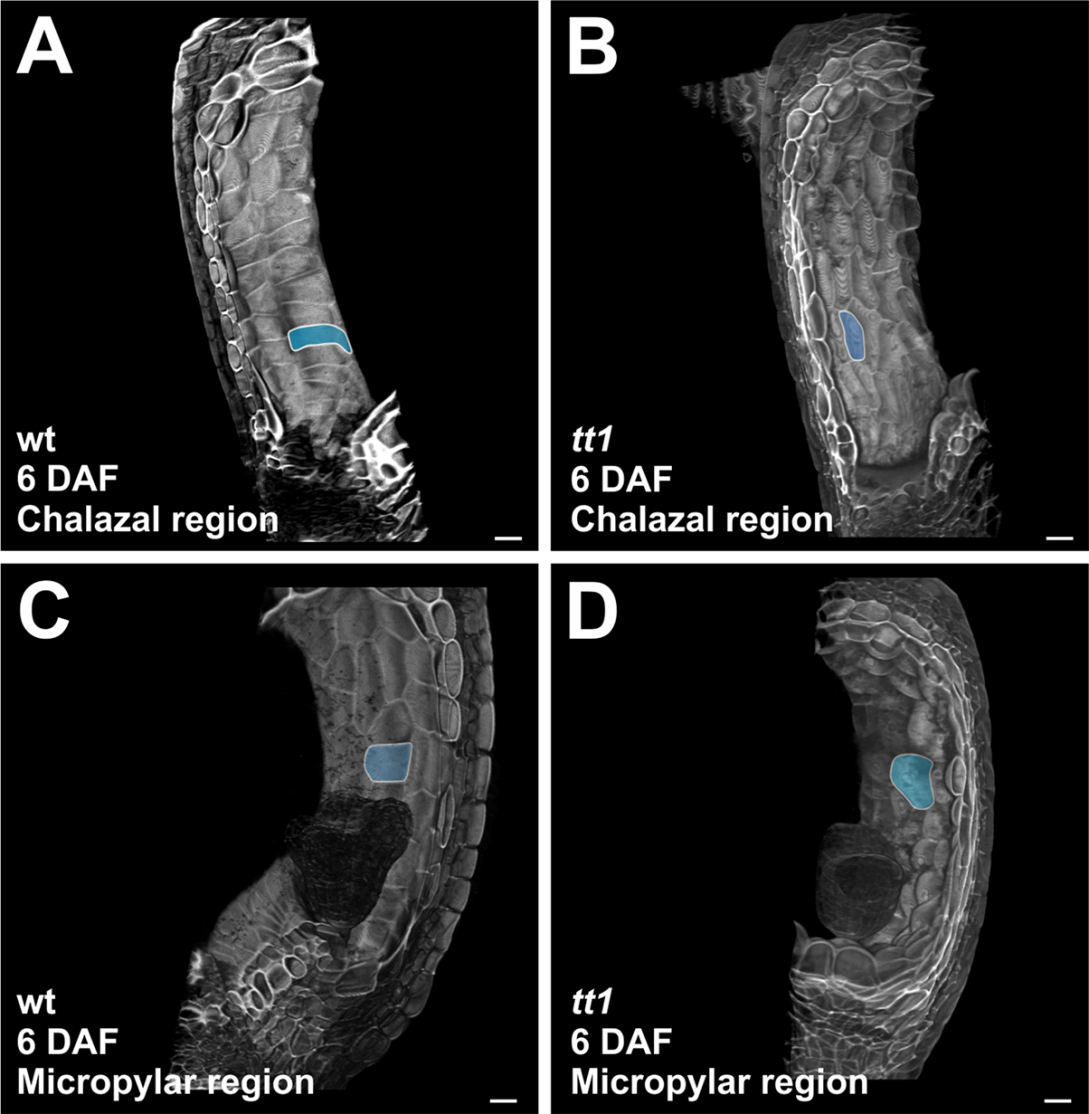
TT1 regulates the polarity of endothelium cell expansion. **(A-D)** Three-dimensionally reconstructed chalazal and micropylar regions of wild type (wt) and *tt1-4* seeds at 6 DAF, imaged using the mPS-PI technique. One endothelium cell per sample is highlighted in blue. Ecotype Ws. Bars = 50 µm.

It has been previously shown that longitudinal mid-planes of *tt16* seeds displayed anticlinally over-elongated endothelium cells, as observed in the chalazal region of the *tt1* endothelium (Nesi et al., 2002; Coen et al., 2017). This *tt16* phenotype is due to the polarity of cell expansion more than to the extent of cell expansion (Coen et al., 2017). Similarly, three-dimensional images revealed that *tt1* chalazal endothelium cells were aligned along the proximal-distal axis of the seed, and thus perpendicular to wild-type cells (**Figures 3A, B**). Therefore, the phenotype of proximal *tt1* endothelium cells observed in two dimensional planes is due to cell orientation defects. By contrast, distal *tt1* endothelium cells appeared periclinally over-expanded, when compared to the wild type (**Figures 3C, D**). Altogether, these data indicate that TT1 modulates the polarity of cell expansion along the proximal-distal axis of the endothelium.

### TRANSPARENT TESTA 1 Regulates Inner Integument 1’ Cell Expansion

**Figure 4:**
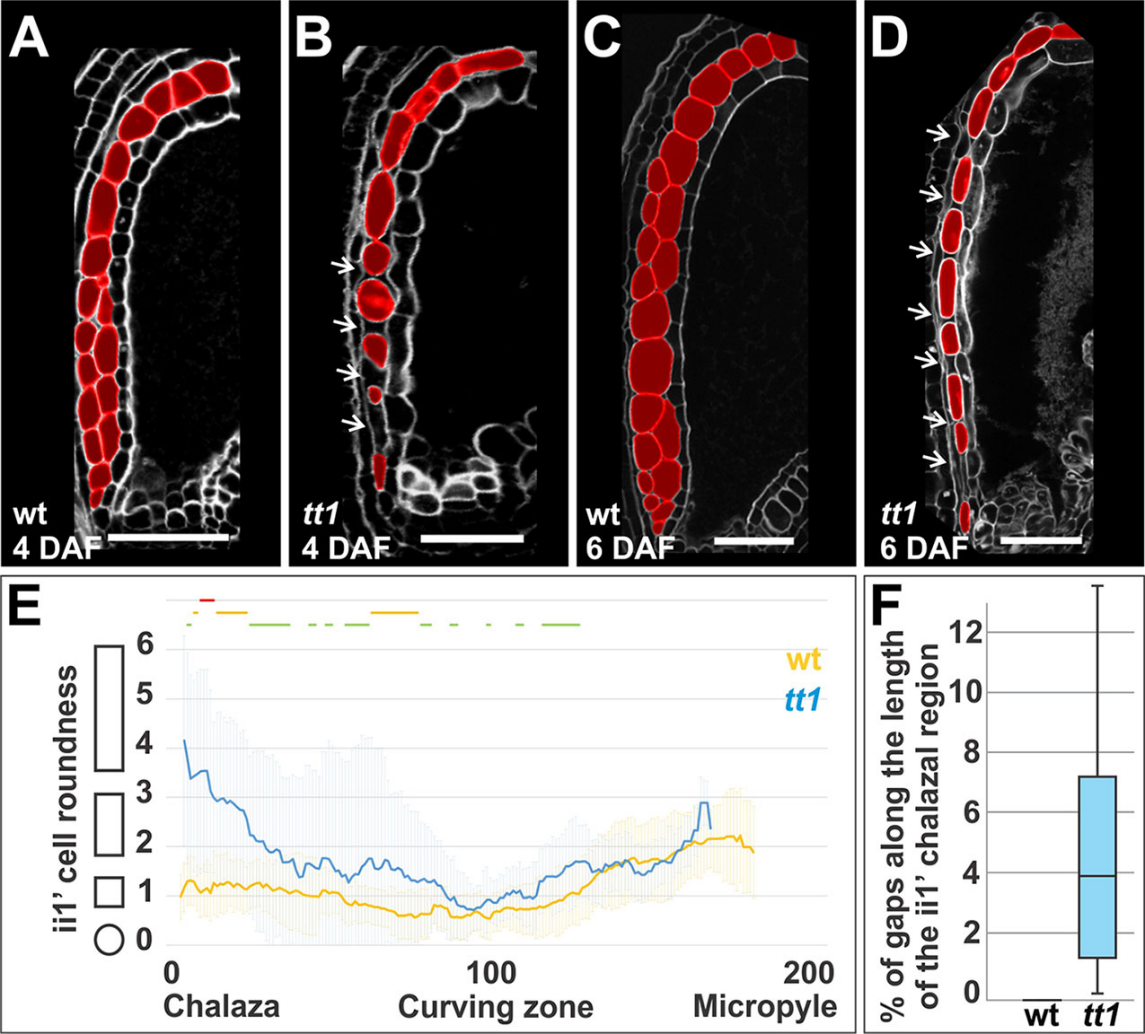
TT1 regulates ii1’ cell expansion. **(A**–**D)** Longitudinal mid-planes of wild type (wt) and *tt1-4* seeds at 4 and 6 DAF, imaged using the mPS-PI technique. ii1’ and ii1’’ cells are highlighted in red. Ecotype Ws. Bars = 50 µm. **(E)** Average ii1’ cell roundness (see *Methods*) along the seed coat proximal-distal axis (arbitrarily divided in 201 points) as observed in longitudinal mid-planes of wild type (wt, blue) and *tt1-4* (yellow) seeds at 4 DAF. The shapes on the left of the graph exemplify how cell shape changes along the *y*-axis. Lines at the top of the graph indicate regions of statistically significant difference between wild type and *tt1-4* (two-tailed Student’s *t*-test; green line: P < 0.05, orange line: P < 0.001, red line: P < 0.00001). Error bars indicate standard deviation (wild type n = 33, *tt1-4* n = 20). Ecotype Ws. **(F)** Box plot representing the percentage of gaps along the length of the ii1’ chalazal region in wild type (wt) and *tt1-4* seeds 4 DAF. (wild type n = 33, *tt1-4* n = 20). Ecotype Ws.

The ii1’ cell layer originates by periclinal cell divisions of the endothelium at the end of ovule development and undergoes severe cell expansion after fertilization (Schneitz et al., 1995; Debeaujon et al., 2003; Coen et al., 2017). To test if TT1 regulates ii1’ development, we studied ii1’ cell parameters in longitudinal mid-planes of wild type and *tt1* seeds at 4 and 6 DAF (**Figures 4A–D**). Quantitative analyses revealed that the shape of ii1’ cells at the chalazal region was significantly more elongated in *tt1* seeds than in wild type seeds, at 4 DAF (**Figure 4E**). Furthermore, some ii1’ cells at the chalazal region appeared disconnected from the others in the *tt1* mutant, a phenotype never observed in the wild type (**Figures 4A–D**). To quantify this latter phenotype, we determined the ratio of total gap length to total length of the ii1’ chalazal region. Although high variability was observed, *tt1* mutant seeds at 4 DAF displayed on average 4.6% of gaps along the length of the ii1’ chalazal region whereas all wild type seeds analyzed showed a continuous ii1’ cell layer (**Figure 4F**). Overall, this analysis indicates that TT1 regulates ii1’ cell expansion.

### TRANSPARENT TESTA 1 Promotes Endothelium and Inner Integument 1’ Cell Division

**Figure 5:**
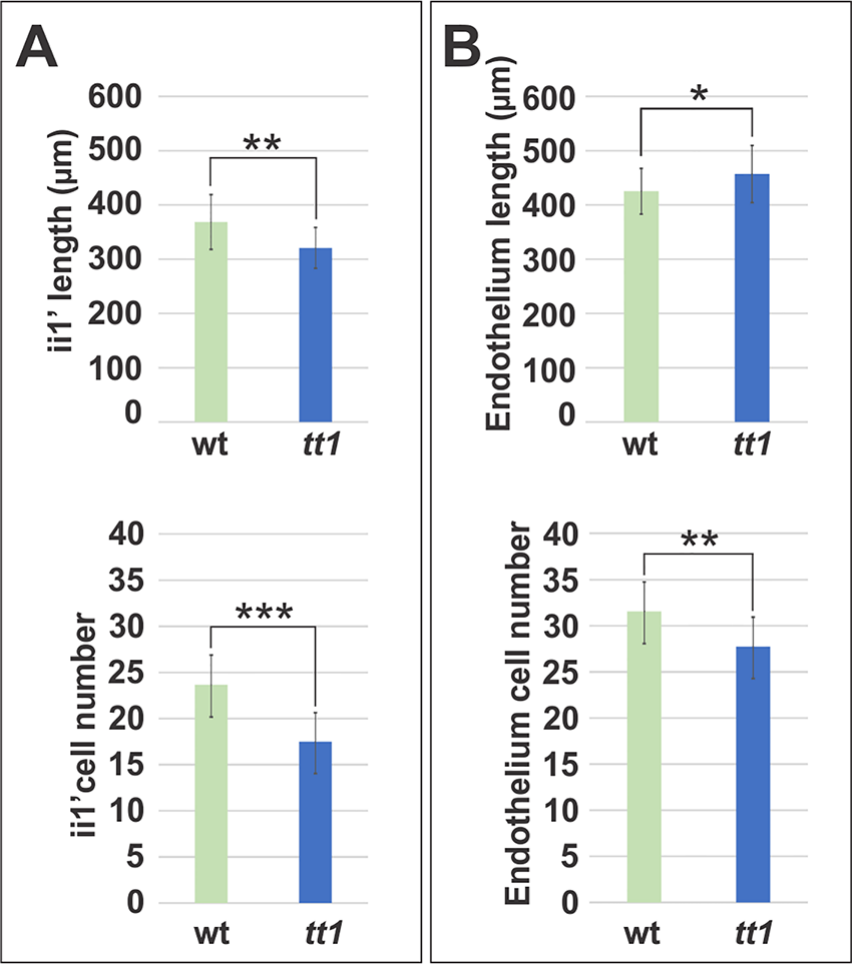
TT1 regulates ii size. **(A**, **B)** Average length and cell number of the gynobasal side of ii1’ **(A)** and endothelium **(B)** cell layers in seed longitudinal mid-planes at 4 DAF. Asterisks indicate statistical significance between wild type and *tt1-4* (two-tailed Student’s *t*-test; *: P < 0.05, **: P < 0.001, ***: P < 0.00001). Error bars indicate standard deviation (wild type n = 33, *tt1-4* n = 20). Ecotype Ws.

To test if the overall size of the ii is affected by the *tt1* mutation, we measured length and cell number of the gynobasal side of endothelium and ii1’ cell layers in seed longitudinal mid-planes. The ii1’ cell layer of *tt1* seeds was shorter and displayed less cells than in the wild type, thus not compensating for reduced cell expansion (**Figure 5A**). By contrast, the *tt1* endothelium appeared slightly longer and counted less cells, when compared to the wild type, thus partially compensating for cell orientation and over-expansion defects (**Figure 5B**). Overall, these data indicate that a size compensation mechanism is put in place only in the endothelium or that TT1 promotes cell division.

### TRANSPARENT TESTA 1 Is Stably Expressed in the Endothelium and Transiently in the Inner Integument 1’

**Figure 6:**
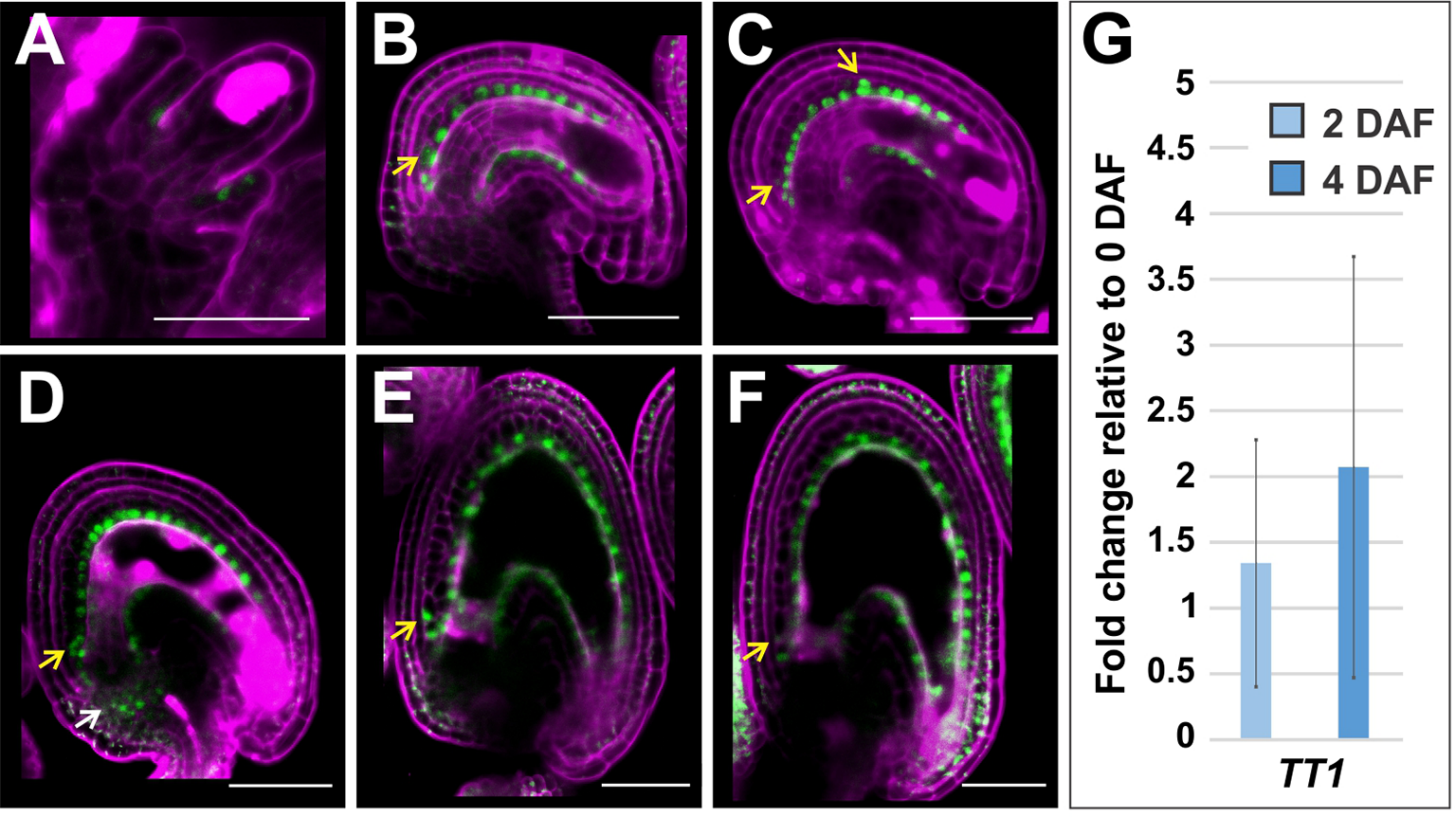
*TT1* expression in ovules and seeds. **(A**–**F)** GFP fluorescence images of *3kbProTT1:gTT1-GFP* ovules at stage 2-V **(A)**, stage 3-VI **(B)**, and maturity **(C)** and seeds at 1 DAF **(D)**, 2 DAF **(E)**, and 3 DAF **(F)**. Yellow and white arrows point to GFP fluorescent ii1’ and chalazal cells, respectively. GFP and propidium iodide fluorescence are in green and purple, respectively. Ecotype Col-0. Bars = 50 µm. **(G)** Quantitative RT-PCR analysis of *TT1* expression in wild type seeds at 2 and 4 DAF versus seeds at 0 DAF. Error bars indicate standard deviation. Expression levels were normalized and averaged from four independent biological samples. Ecotype Col-0.

*TT1* promoter analyses indicated that *TT1* is expressed in the endothelium and, to a lesser extent, in the other integument cell layers (; ). RNA in situ hybridization experiments showed *TT1* expression in the endothelium but high background signal levels did not allow to conclusively address *TT1* expression in other integument cell layers (). The promoter region of *CmWIP1*, *TT1* orthologue in melon, contains retrotransposons whose methylation regulates CmWIP1 expression (Coen et al., 2019). Likewise, the promoter region of *TT1* is annotated as carrying copia-like retrotransposons and RC/Helitron and DNA/MuDR transposon fragments, thus suggesting that its expression might also be regulated by cis-epigenetic mechanisms. Since previous studies included a relatively small *TT1* promoter sequence downstream of the transposon region and to account for the possibility of TT1 post-translational regulation, we created a marker line carrying *TT1* 3 kb promoter region (including part of the transposon region) upstream of *TT1* genomic sequence translationally fused to *GFP* (*3kbProTT1:gTT1-GFP*). Seven independent *3kbProTT1:gTT1-GFP* lines showed fluorescence in the nuclei of endothelium cells from stage 2-IV of ovule development (Schneitz et al., 1995) till globular embryo stage of seed development (**Figures 6A–F**). In newly periclinally divided endothelium cells we observed fluorescence both in endothelium and ii1’ cells (**Figure 6C**). Furthermore, we detected GFP fluorescence in the most proximal ii1’ cells (**Figures 6B–F**) and in a subdomain of the seed chalaza (**Figure 6D**). Non-nuclear auto-fluorescence signal is also visible in the seed coat (**Figures 6B–E**). Overall, these data confirm *TT1* expression in the endothelium and suggest a novel layer of transcriptional and/or post-transcriptional regulation in the other integument cells layers and in the chalaza, when compared to previously published data (Sagasser et al., 2002; Coen et al., 2019). Finally, we did not detect any significant change in *TT1* expression across fertilization by RT-qPCR analyses (**Figure 6G**).

### TRANSPARENT TESTA 1 Acts Downstream of TRANSPARENT TESTA 16 At the Chalazal Region

**Figure 7:**
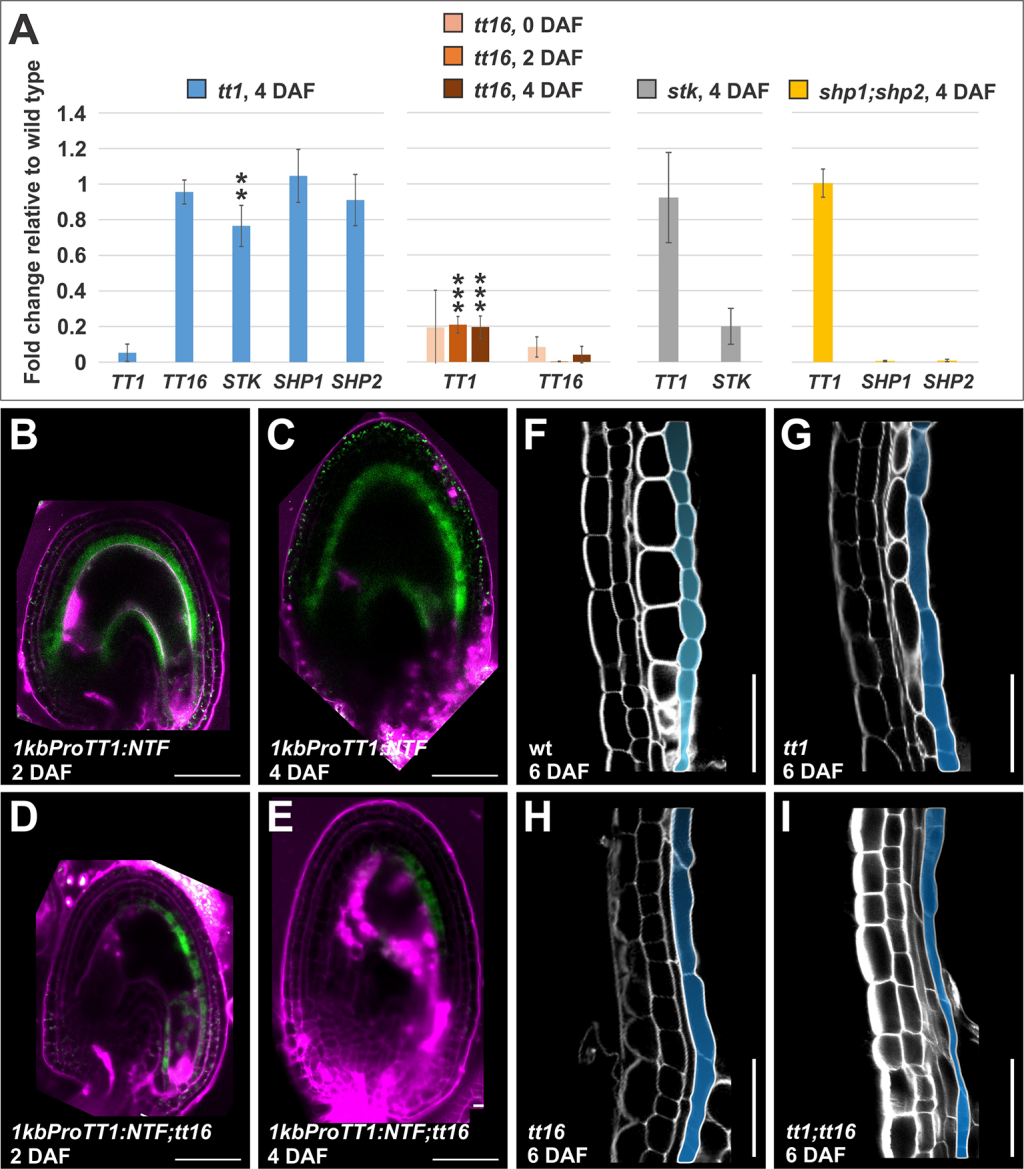
TT16 regulates *TT1* expression. **(A)** Quantitative RT-PCR analyses of *TT1*, *TT16*, *STK*, *SHP1*, and *SHP2* expression in *tt1-3*, *tt16-1*, *stk-2*, and *shp1-1;shp2-1* mutant seeds relative to wild type. Asterisks indicate statistical significance between wild type and mutant (two-tailed Student’s *t*-test; **: P < 0.001, ***: P < 0.00001). Error bars indicate standard deviation. Expression levels were normalized and averaged from four independent biological samples. Ecotype Col-0. **(B**–**E)** GFP fluorescence images of *1kbProTT1:NTF* **(C**, **D)** and *1kbProTT1:NTF*;*tt16-1* **(E**, **F)** seeds at 2 **(C**, **E)** and 4 **(D**, **F)** DAF. GFP and propidium iodide fluorescence are in green and purple, respectively. Ecotype Col-0. Bars = 50 µm. **(F**–**I)** Longitudinal mid-planes of integument cells at the chalazal area of wild type (wt) **(G)**, *tt1-4* **(H)**, *tt16-1* **(I)**, and *tt1-4;tt16-1* **(J)** seeds at 6 DAF, imaged using the mPS-PI technique. Endothelium cells are highlighted in blue. Ecotype Ws. Bars = 50 µm.

The MADS box transcription factor genes *TT16*, *STK*, *SHP1*, and *SHP2* are known to affect the development of the ii (Nesi et al., 2002; Mizzotti et al., 2012; Mizzotti et al., 2014; Ehlers et al., 2016; Coen et al., 2017). To determine their genetic interactions with *TT1*, we tested their expression levels in each other mutant background relative to wild type. Whereas *TT16*, *SHP1*, and *SHP2* expression was not affected by the *tt1* mutation, *STK* expression was mildly downregulated in *tt1* seeds at 4 DAF, when compared to the wild type (**Figure 7A**). Conversely, *TT1* mRNA levels were unchanged in *stk* and *shp1*;*shp2* mutants while drastically down-regulated in *tt16* seeds at 4 DAF, when compared to the wild type (**Figure 7A**).

TT16 has been shown to have fertilization-dependent functions, such as nucellus elimination and PA deposition (Nesi et al., 2002; Xu et al., 2016). To test if TT16 regulates *TT1* expression in a fertilization-dependent manner, we analyzed wild type and *tt16* ovules (0 DAF) and seeds (2 DAF). *TT1* expression was equally reduced before and after fertilization in the *tt16* mutant when compared to the wild type (**Figure 7A**).

To thoroughly characterize TT16 role in regulating *TT1* expression, we introgressed the *1kbProTT1:NTF* marker line (Coen et al., 2019), coding for a GFP chimeric protein that binds to the nuclear membrane (Deal and Henikoff, 2011) under the control of the *TT1* 1 kb promoter region, in a *tt16* mutant background. Whereas we detected GFP fluorescence all along the endothelium of *1kbProTT1:NTF* seeds (**Figures 7B, C**), the GFP signal was absent in the chalazal region of the endothelium of *1kbProTT1:NTF;tt16* seeds (**Figures 7D, E**). Non-nuclear auto-fluorescence signal is also visible in the seed coat (**Figures 7B–E** and **Supplemental Figure 2**). Altogether, these results suggest that TT16 positively regulates *TT1* expression solely in chalazal endothelium cells.

In line with our transcriptional analyses, longitudinal mid-planes of *tt1* and *tt16* seeds displayed equally over-elongated endothelium cells at the chalazal region when compared to the wild type (**Figures 7F–H**). To further characterize *TT1* and *TT16* genetic interaction, we created a *tt1;tt16* double mutant. The chalazal region of the *tt1;tt16* inner integument displayed over-elongated endothelium cells as observed in either single mutant line (**Figure 7G–I**). Overall, these data indicate that *TT1* is epistatic to *TT16* in endothelium cell development at the chalazal region.

### TRANSPARENT TESTA 1 Downstream Target Genes

**Figure 8:**
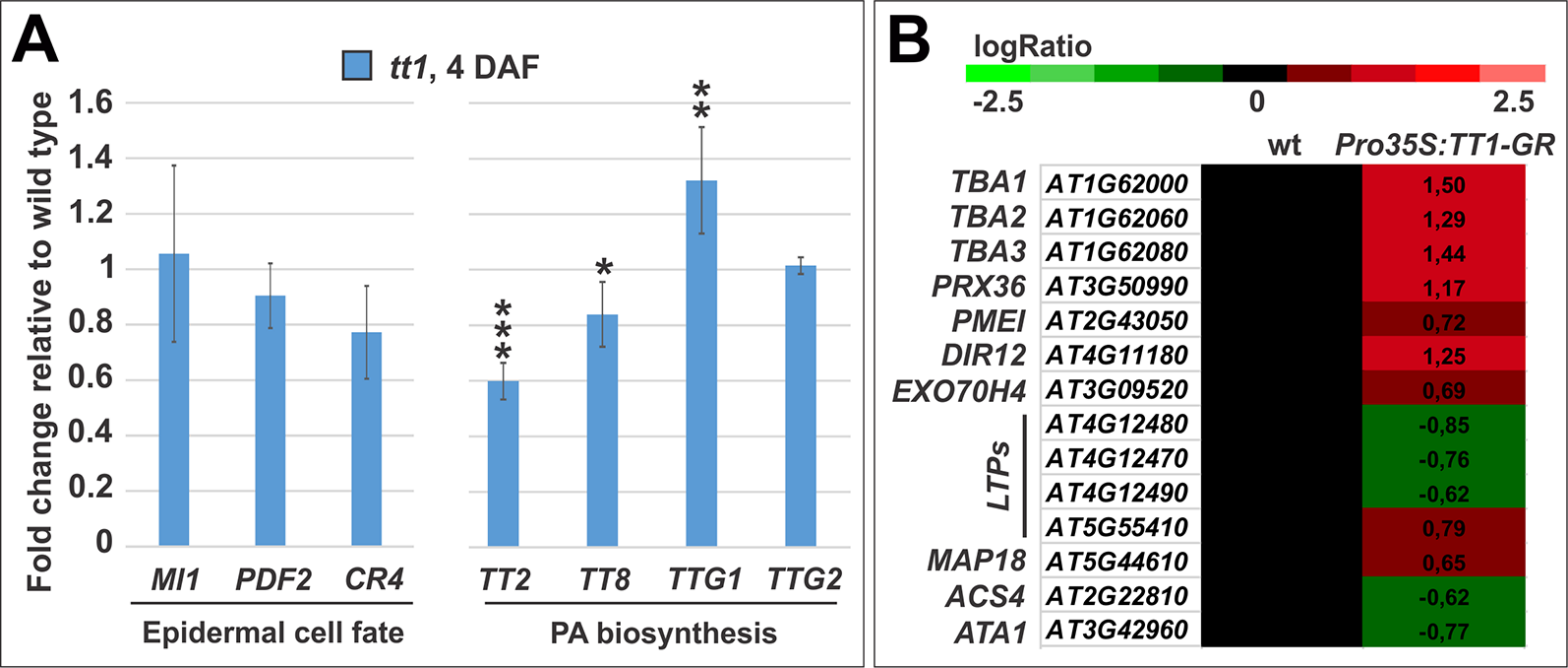
TT1 downstream target genes **(A)** Quantitative RT-PCR analyses of *ML1*, *PDF2*, *CR4*, *TT2*, *TT8*, *TTG1*, and *TTG2* expression in *tt1-3* mutant seeds relative to wild type. Asterisks indicate statistical significance between wild type and mutant (two-tailed Student’s *t*-test; *: P < 0.05, **: P < 0.001, ***: P < 0.00001). Error bars indicate standard deviation. Expression levels were normalized and averaged from four independent biological samples. Ecotype Col-0. **(B)** Fold change values in gene expression between mock and DEX treated siliques in wild type (wt) and *35S:TT1-GR* backgrounds as detected by microarray analyses. Ecotype Col-0.

Our analysis of *TT1* expression pattern and loss of function mutant phenotypes suggest a role for TT1 in establishing endothelium cell identity. The endothelium is an epidermal cell layer (Schneitz et al., 1995) and expresses the *MERISTEM LAYER 1* (*ML1*) (**Supplemental Figure 3**), *PROTODERMAL FACTOR 2* (*PDF2*), and *CRINKLY4* (*CR4*) epidermal cell fate genes (Lu et al., 1996; Sessions et al., 1999; Abe et al., 2003; Gifford et al., 2003; San-Bento et al., 2014; Huang et al., 2016). Nevertheless, *ML1*, *PDF2*, and *CR4* expression was not affected by the *tt1* mutation, when compared to the wild type (**Figure 8A**).

The *TT1* gene has been first identified for its loss of function transparent TESTA phenotype (Sagasser et al., 2002) and has been shown to positively regulate PA biosynthetic genes (Appelhagen et al., 2011). To further characterize its role in PA biosynthesis, we tested the expression of key regulatory genes of the PA pathway in the *tt1* mutant. *TT2*, *TT8*, *TRANSPARENT TESTA GLABRA 1* (*TTG1*), and *TTG2* encode for transcription factors that regulate the flavonoid late metabolism in developing siliques. *TT2* and *TT8* were down-regulated whereas *TTG1* up-regulated in *tt1* seeds at 4 DAF, when compared to the wild type (**Figure 8A**).

To identify novel TT1 downstream target genes, we created an inducible TT1 transcription factor fused to the rat glucocorticoid receptor (GR) under the control of the constitutive cauliflower mosaic virus *35S* promoter (*Pro35S:TT1-GR*). Dexamethasone (DEX) treatment releases the GR transcription factor chimeric protein from a cytoplasmic HEAT SHOCK PROTEIN 90 complex that prevents its nuclear translocation and therefore its functionality (Schena et al., 1991). Addition of DEX to *Pro35S:TT1-GR* plants caused leaf elongation and narrowing similar to plants that overexpress *TT1* (Sagasser 2002), indicating that the TT1-GR chimeric protein retains function. To minimize sample handling, we analyzed entire inflorescences bearing ovules and seeds up to the early torpedo embryo stage. To prevent indirect transcriptional effects of the inducible TT1-GR protein, we infiltrated *Pro35S:TT1-GR* inflorescences with cycloheximide (CHX), an inhibitor of protein synthesis. We then treated the samples with DEX or a mock solution for 1 h. To discount for the unspecific effect of DEX, we conducted an identical experiment with wild type inflorescences. Transcript levels across the entire genome were measured by hybridization to CATMA microarrays (Allemeersch et al., 2005). To increase our ability to find genuine targets (i.e., decrease false negatives) and reduce the background of false positives, we concentrated on genes that showed a statistically significant interaction between time of treatment and genotype. This approach identified 74 up-regulated and 16 down-regulated genes by DEX induction in the *Pro35S:TT1-GR* line, which were unaffected in the wild type background (**Supplemental Table 1**). To investigate the range of processes that could be regulated by TT1, we performed a GO annotation analysis. We detected response to stress and stimulus, developmental processes, protein metabolism, cell organization and biogenesis, signal transduction, transport, and transcription DNA-dependent categories.

A GO enrichment analysis for biological processes revealed significant enrichments in GO annotations in mucilage biosynthetic process and seed coat development. Among the proteins regulated by TT1-GR, we detected a known mucilage extrusion factor, PEROXIDASE 36 (PRX36) (Kunieda et al., 2013), the DIRIGENT 12 (DIR12) protein, which is a member of the *PRX36* co-expression network (Ranocha et al., 2014), three abundant mucilage proteins, TESTA ABUNDANT 1 (TBA1), TBA2, and TBA3 (Tsai et al., 2017), a mucilage related (Voiniciuc et al., 2015) PECTIN METHYL ESTERASE INHIBITOR (PMEI), which promotes mucilage release (Saez-Aguayo et al., 2013), and the EXOCYST SUBUNIT EXO70 FAMILY PROTEIN H4 (EXO70H4), a subunit of the exocyst complex that participates in mucilage deposition (Kulich et al., 2010) (**Figure 8B**).

A GO enrichment analysis for cellular components showed an enrichment in apoplastic proteins. Among the genes annotated to be secreted, we found that TT1-GR regulates the expression of four *LIPID TRANSFER PROTEIN* (*LTP*) genes (**Figure 8B**). LTPs have been implicated in cuticle formation and cell wall extension (Salminen et al., 2016), which are both processes regulated by TT1 (Loubery et al., 2018; Coen et al., 2019).

Validation of our microarray analysis comes from the finding of the only experimentally verified TT1 downstream target gene. *CmWIP1*, *TT1* orthologue in melon, has been shown to repress the expression of the melon *1-AMINOCYCLOPROPANE-1-CARBOXYLIC ACID SYNTHASE 7* (*ACS7*), an ethylene biosynthetic enzyme that regulates sex determination. In line with this study, we revealed that TT1-GR represses *ACS4*, an orthologue of *ACS7*, and regulates the expression of four ethylene responsive factor genes (**Figure 8B** and **Supplemental Table 1**). Furthermore, TT1 downregulated the expression of *ARABIDOPSIS TAPETUM 1* (*ATA1*), the orthologue of the *TASSLE SEED 2* gene that controls sex determination in maize (DeLong et al., 1993) (**Figure 8B**). By contrast, we did not detect PA regulatory or biosynthetic genes.

Finally, to find candidate genes possibly responsible for the cell expansion phenotype of *tt1* endothelium cells, we looked for target genes annotated as cell shape regulators. This screen retrieved only the MICROTUBULE-ASSOCIATED PROTEIN 18 (MAP18) (also known as PLASMA MEMBRANE ASSOCIATED CA2+-BINDING PROTEIN-2, PCaP2), which regulates directional cell growth and cortical microtubule organization (**Figure 8B**) (Wang et al., 2007; Kato et al., 2019). According to the laser microdissection transcriptomics data by Le and coworkers (Le et al., 2010), *MAP18* is expressed in the seed coat at the linear cotyledon stage. Nevertheless, *map18* mutant seeds displayed wild type looking endothelium cells (**Supplemental Figure 1**).

## Discussion

The seed coat, as a whole, is a highly polar structure. Nevertheless, less is known about the development of polar axes in individual integument cell layers. Here, we characterize the role of the Arabidopsis TT1 transcription factor in modulating polarity along the proximal-distal axis of the endothelium and shed light on TT1 upstream and downstream regulatory pathways.

### TRANSPARENT TESTA 1 and TRANSPARENT TESTA 16 Modulate Cell Expansion Along the Endothelium Proximal-Distal Polarity Axis

**Figure 9:**
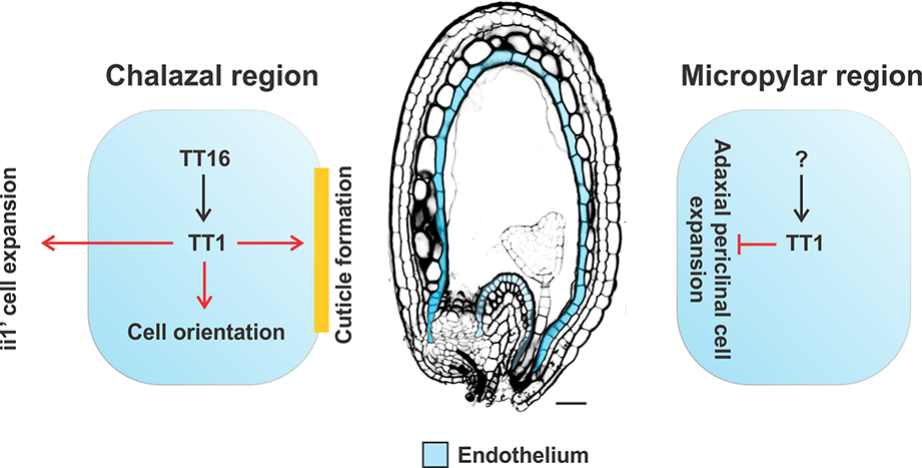
Model for the regulation of integument development by TT1. Black and red arrows indicate transcriptional and functional relationships, respectively.

We identified proximal-distal polarity along the wild type endothelium and showed that it is severely affected by the *tt1* mutation. At the chalazal region, *tt1* endothelium cells appeared oriented along the proximal-distal axis, perpendicularly to wild type cells. By contrast, *tt1* endothelium cells at the micropylar region were more periclinally expanded on their adaxial side. These data indicate that TT1 regulates both orientation and extent of cell expansion (**Figure 9**). Morphological defects of *tt1* seeds as well as *TT1* early expression in ovules favor the hypothesis that TT1 regulates endothelium development and only indirectly PA biosynthesis. In line with this hypothesis, we detected lower expression of PA regulatory genes in *tt1* seeds, possibly responsible for the down regulation of PA biosynthetic genes previously described in *tt1* seeds (Appelhagen et al., 2011), but we did not find either category of genes in transcriptomic analyses aimed at revealing TT1 immediate target genes. Alternatively, PA biosynthetic genes might be regulated by TT1-TT2 protein complexes, as suggested by Appelhagen and coworkers (Appelhagen et al., 2011), thus requiring the induction of both transcription factors to affect PA biosynthesis.

We discovered that TT16 promotes *TT1* expression in the chalazal but not micropylar region of the endothelium. In agreement with this result, *tt16* and *tt1* seeds show the same lack of cuticle and cell orientation phenotypes in the chalazal endothelium whereas opposite phenotypes in the micropylar region (**Figure 9**) (Nesi et al., 2002; Coen et al., 2017; Coen et al., 2019). We hypothesize that a different transcriptional regulator might control *TT1* expression in the micropylar region of the endothelium (**Figure 9**). Downstream of TT1, we discovered *MAP18* as putative target gene responsible for endothelium cell polar growth. MAP18 has been shown to contribute to directional cell growth and cortical microtubule organization (Wang et al., 2007; Kato et al., 2019). Nevertheless, *map18* seeds did not display any obvious mutant phenotype possibly because of gene redundancy. Furthermore, TT1 affected the expression of a number of genes involved in the ethylene pathway, as previously shown in melon (Martin et al., 2009). Ethylene regulates anisotropic cell growth and might therefore be responsible for *tt1* cell expansion phenotypes (Kieber et al., 1993; Rodrigues-Pousada et al., 1993; Xu et al., 2008). Finally, our microarray data revealed LTP genes as putative *TT1* downstream targets. The tobacco NtLTP1.6 has been found to regulate cell wall extension, thus suggesting that LTPs might affect endothelium cell elongation. LTPs play also a role in intracuticular or epicuticular wax deposition (Cameron et al., 2006). The adaxial side of the endothelium is covered by a cuticle layer that lacks or is low in waxes (Coen et al., 2019). TT1, which has been shown to promote cuticle deposition (Loubery et al., 2018; Coen et al., 2019), might therefore prevent wax accumulation by repressing *LTPs* expression. The cuticle creates a mechanical barrier between the endothelium and the endosperm that might repress cell expansion and be partly responsible for the flat adaxial surface of wild type endothelium cells. Nevertheless, *tt16* endothelium cells, which display a more dramatic cuticle phenotype than *tt1*, do not expand periclinally as in *tt1* seeds, indicating that the cuticle alone is not enough to explain the squared shape of wild type endothelium cells (Coen et al., 2017; Coen et al., 2019).

We hypothesize that changes in cell size, shape, and orientation along the endothelium proximal-distal axis might be necessary to achieve proper seed shape. In line with this interpretation, the chalazal region of *tt1* seeds is flatter than that of wild type seeds, a phenotype also observed in *tt16* seeds (Nesi et al., 2002). Furthermore, reduced periclinal cell expansion at the endothelium micropylar region might be important to create an empty groove that allows the correct growth of the embryo. In the *tt16* mutant, the ii1’ cell layer extends toward the micropyle, thus narrowing the micropylar region and preventing, in some extreme cases, embryo growth (Coen et al., 2017). Similarly, TT1 might prevent the expansion of micropylar endothelium cells to facilitate embryo development. On the other hand, the seeds of a number of plant species, whose micropylar end is formed by the ii and not the oi as in Arabidopsis, show endothelium cell thickening as a way to restrict the micropylar pore (Coen and Magnani, 2018). Further evidence for the importance of an endothelium proximal-distal polarity axis comes from the lack of cuticle at the seed micropylar end, which has been suggested to allow diffusion of nutrients and developmental signals from zygotic to maternal tissues (Loubery et al., 2018; Coen et al., 2019). We, therefore, speculate that regulation of TT1 function or expression might be responsible for such natural morphological diversity.

### TRANSPARENT TESTA 1 Promotes Inner Integument 1’ Cell Expansion and Fertilization Responsiveness

The ii’ cell layer is the only integument cell layer that does not respond to the endosperm signal that relieves the repressive action of the FERTILIZATION INDEPENDENT ENDOSPERM (FIE) and MULTICOPY SUPPRESSOR OF IRA1 (MSI1) Polycomb Group (PcG) proteins, which prevent the fertilization-independent expansion of the integuments (Roszak and Kohler, 2011). A fraction of unfertilized *fie/+* and *msi1/+* ovules develop into enlarged autonomous seeds that exhibit a developed seed coat, which accumulates PAs, and a degenerated nucellus, both hallmarks of fertilization (Roszak and Kohler, 2011; Xu et al., 2016). However, *fie/+* and *msi1/+* enlarged autonomous seeds display an underdeveloped and discontinuous ii1’ cell layer made of unexpanded cells and empty spaces (Coen et al., 2017; Fiume et al., 2017b), as observed in *tt1* seeds. These data suggest that TT1 promote FIE- and MSI1-independent expansion of ii1’ cells.

The ii1’ cell layer differs from the other cell layers for its sub-epidermal position and origin by periclinal cell divisions. It has been previously shown that sub-epidermal outer integument cell stripes, developing in a fraction of wild type seeds, respond to the FIE and MSI1 repressive mechanism as epidermal integument cell layers (Fiume et al., 2017a). This study suggests that the sub-epidermal position of the ii1’ is not sufficient to explain for its insensitivity to FIE and MSI1 and points to an asymmetric periclinal cell division of the endothelium as a more likely scenario. Our transcriptional analysis showed a more restricted and transient expression of *TT1* in the ii1’ cell layer than previously described (Sagasser et al., 2002; Coen et al., 2019). We detected *TT1* expression solely in newly developed ii1’ cells, right after endothelium periclinal cell division, and in the most proximal ii1’ cells. Compared to previous analyses, we tested a longer promoter region containing transposon sequences, which have been shown to regulate *TT1* orthologue expression in melon (Martin et al., 2009), and *TT1* genomic sequence. These data suggest that Arabidopsis *TT1* expression in the ii1’ might be negatively regulated by transposons or *TT1* intronic sequence. In an alternative non-exclusive scenario, TT1 protein might be rapidly degraded in the ii1’ cell layer. TT1 protein pattern across fertilization, constantly present in the endothelium while only transiently in most ii1’ cells, might explain for the ii1’ unique responsiveness to fertilization.

Our data show that *TT1* expression in the ii1’ is activated by TT16. It has been shown that TT16 regulates ii1’ cell expansion and patterning along the proximal-distal axis (Mizzotti et al., 2012; Coen et al., 2017). In line with these results, longitudinal mid-planes of both *tt1* and *tt16* seeds display ii1’ cells with an anticlinally over-elongated shape when compared to the wild-type (Coen et al., 2017). Nevertheless, *tt16* seeds do not display empty spaces in between ii1’ cells, thus suggesting that TT1 and TT16 also play independent functions as observed in the endothelium micropylar region.

## Data Availability Statement

The datasets generated for this study can be found in the Microarray data from this article were deposited in the international repository GEO, Gene Expression Omnibus (Edgar et al., 2002), accession number: GSE 134014. All steps of the experiment, from growth conditions to bioinformatic and statistical analyses, were detailed in CATdb (Gagnot et al., 2008) (Project: RA15-05_TT1) according to the “Minimum Information About a Microarray Experiment” standards.

## Author Contributions

OC performed the research, analyzed the data, and helped to write the article. JL, WX, and DG helped to perform morphological analyses, SP performed the transcriptomic analysis and CP performed the transmission electron microscopy analysis. LL helped to analyze the data and write the article. EM designed the research and wrote the article.

## Conflict of Interest

The authors declare that the research was conducted in the absence of any commercial or financial relationships that could be construed as a potential conflict of interest.
