# Supplementary material for: A TRANSPARENT TESTA Transcriptional Module Regulates Endothelium Polarity
Source: Front Plant Sci. 2020 Feb 6;10:1801. doi: 10.3389/fpls.2019.01801 (PMC7015901; doi:10.3389/fpls.2019.01801)
Supplement: Supplementary file 1 [file DataSheet_1.docx]

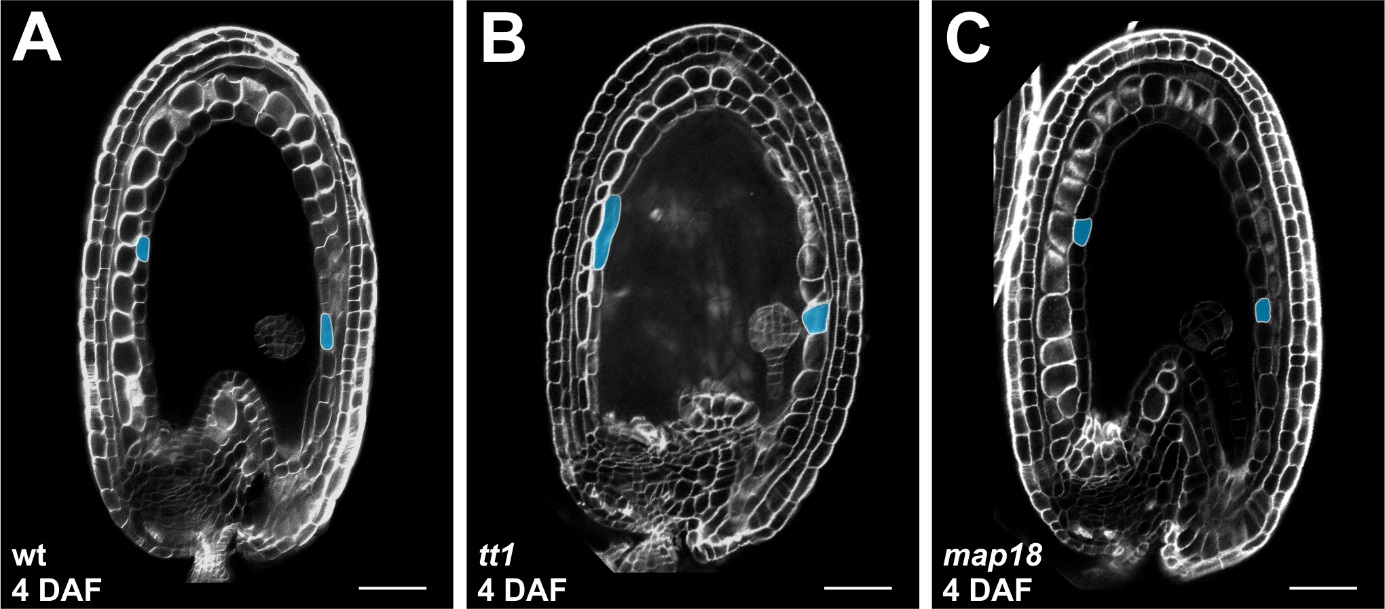


**Supplemental Figure 1.**

**TT1 regulates endothelium cell expansion**

**(A)** to **(C)** Longitudinal mid-planes of wild type (wt, A), *tt1-3* (B) and *map18* (*pcap2* allele, C) seeds at 4 DAF, imaged using the calcofluor staining imaging technique. Two endothelium cells per sample are highlighted in blue. Ecotype Col-0. Bars = 50 µm.


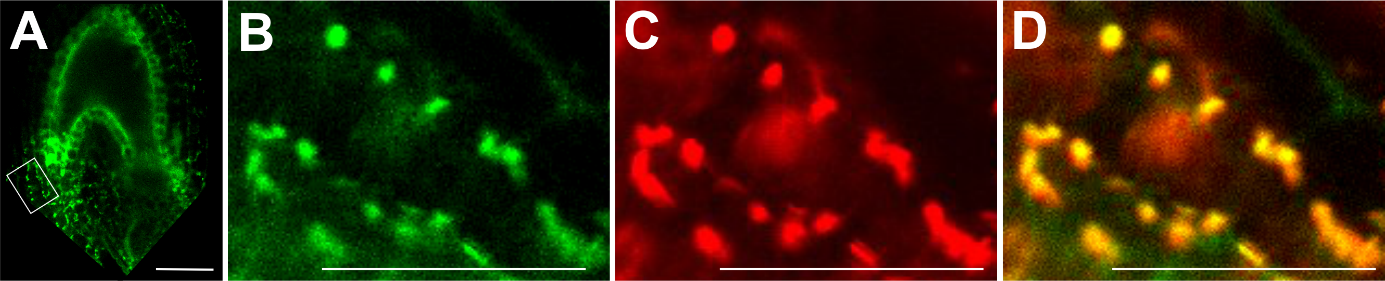


**Supplemental Figure 2.**

**Non-nuclear auto-fluorescence**

**(A)**GFP fluorescence image of a *1kbProTT1:NTF* seed at 4 DAF*.* Bars = 50 µm. **(B)**to**(D)**Close-up GFP (B), auto-fluorescence (650-750 nm) (C), and GFP-auto-fluorescence superimposed (D) fluorescence images of the white rectangle in (A). Bars = 25 µm.


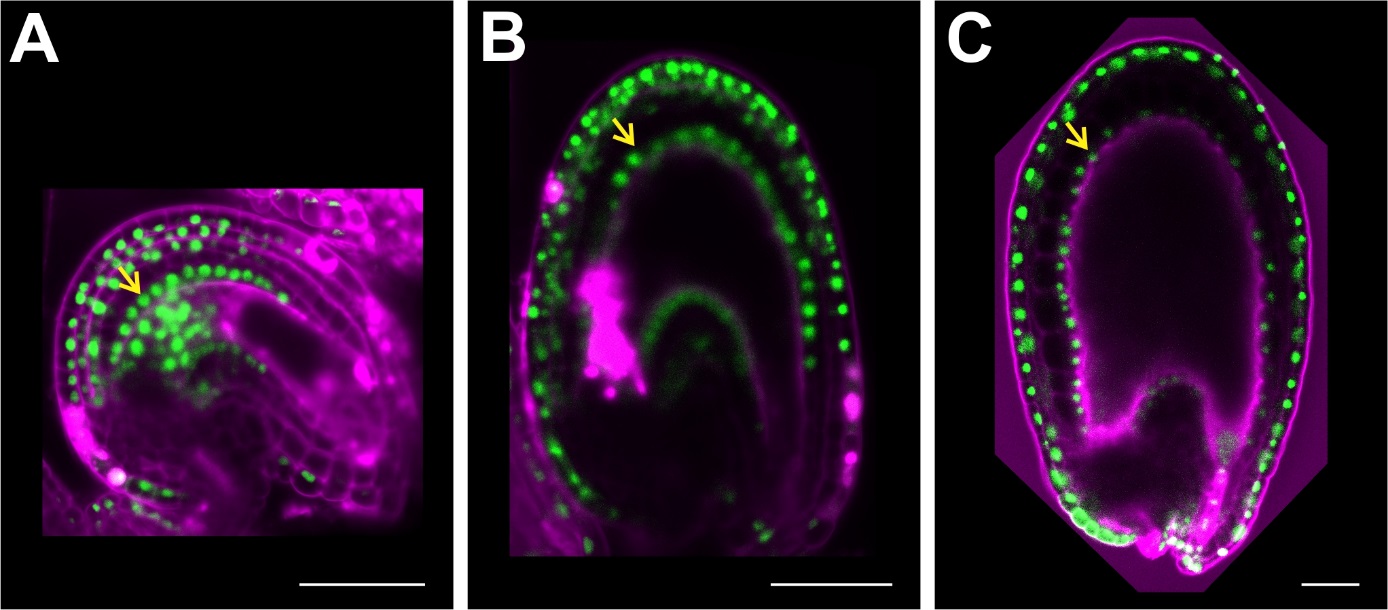


**Supplemental Figure 3.**

***ML1* is expressed in the endothelium**

**(A)**to**(C)**mCitrine fluorescence images of *ProML1:gML1-mCitrine;ml1-3* ovules at stage 2-V (A), and seeds at 2 DAF (B) and 4 DAF (C) Yellow arrows point to mCitrine fluorescent ii1/endothelium cells. mCitrine and propidium iodide fluorescence are in green and purple, respectively. Ecotype Col-0. Bars = 50 µm.

**Supplemental Table 1.**

**Primers used for Quantitative PCRs analyses**

| *GAPDH-Q-F* | GGTACGACAACGAATGGGGT | *GAPDH-Q-R* | TGACTGCGCATGGAATCAGT | |
| --- | --- | --- | --- | --- |
| *AT3G25800-Q-F* | AATCGGTTGTGGAGAAGACG | *AT3G25800-Q-R* | GCGAAAAACCTGACATCAACAT | |
| *AT4G02080-Q-F* | GCTGTGTTATTATTAAGCCGTAAG | *AT4G02080-Q-R* | AAAGCTAGGTACGGTTTAAGAC | |
| *AT4G12590-Q-F* | GAGATGAAAATGCCATTGATGAC | *AT4G12590-Q-R* | GCACCCAGACTCTTTGATG | |
| *TT1-Q-F* | GGATCCCTTGTTACTGCTGC | *TT1-Q-R* | TCTTCTCATGTGTTCGCCAATC | |
| *TT16-Q-F* | GATTGCGACTTCCTGATCATC | *TT16-Q-R* | AGTCCGTCAAGCTCATTAGG | |
| *ML1-Q-F* | TCACACACAACGCCAGATTC | *ML1-Q-R* | GCGACTCAGCTCCTTTCTTTG | |
| *PDF2-Q-F* | GCAACGCCAAATTCAAGAGC | *PDF2-Q-R* | TCACGGCTCAACTCTTTTCG | |
| *CR4-Q-F* | CCTGTGAAATCAGAGGCAGA | *CR4-Q-R* | CTCTATCCGTCGTGCACAGT | |
| *TT2-Q-F* | TCACTTCCCGGATTTGATGGA | *TT2-Q-R* | GAGCCAATCTTCATCGTCGC | |
| *TT8-Q-F* | AGGAAATGAGGCTTGGCTCT | *TT8-Q-R* | TGTGGGGTGTGACATGAGAA | |
| *TTG1-Q-F* | CAGTCCTCCTTCTCTCCGTC | *TTG1-Q-R* | GTTTCGGCTCTACATCGTTCC | |
| *STK-Q-F* | GCACTGTCCAAGAAATCAATGC | *STK-Q-R* | TAGCTTTCTCAAGGCGATTCTC | |
| *SHP1-Q-F* | GGAATCGAGTGTGATACAAGG | *SHP1-Q-R* | ATTCGGTTCAAGAAGGTTCAC | |
| *SHP2-Q-F* | TTAACCCTCCGACCATCACC | *SHP2-Q-R* | GAATGTCCCGAATCTGTCTCC | |
|  | **Average melting temperature (^o^C)** | **Standard deviation** | |  |
| *TT1* | 84,72 | 2,69 | |  |
| *TT16* | 82,67 | 0,25 | |  |
| *STK* | 81,33 | 0,24 | |  |
| *SHP1* | 80,50 | 0,00 | |  |
| *SHP2* | 81,36 | 1,65 | |  |
| *ML1* | 82,50 | 0,00 | |  |
| *PDF2* | 79,10 | 0,57 | |  |
| *CR4* | 80,04 | 0,14 | |  |
| *TT2* | 81,75 | 0,61 | |  |
| *TT8* | 79,82 | 0,24 | |  |
| *TTG1* | 84,93 | 0,18 | |  |
| *TTG2* | 82,78 | 0,26 | |  |
| *GADPH* | 82,38 | 0,23 | |  |
| *AT5G12590* | 79,63 | 0,22 | |  |
| *AT4G02080* | 77,84 | 0,24 | |  |
| *AT5G25800* | 81,91 | 0,20 | |  |
